# Effect of the IL-1 Receptor Antagonist Kineret^®^ on Disease Phenotype in *mdx* Mice

**DOI:** 10.1371/journal.pone.0155944

**Published:** 2016-05-23

**Authors:** Margaret E. Benny Klimek, Arpana Sali, Sree Rayavarapu, Jack H. Van der Meulen, Kanneboyina Nagaraju

**Affiliations:** 1 Research Center for Genetic Medicine, Children’s National Medical Center, Washington, District of Columbia, United States of America; 2 Department of Integrative Systems Biology, The George Washington University, Washington, District of Columbia, United States of America; University of Louisville School of Medicine, UNITED STATES

## Abstract

Duchenne muscular dystrophy (DMD) is an X-linked muscle disease caused by mutations in the dystrophin gene. The pathology of DMD manifests in patients with progressive muscle weakness, loss of ambulation and ultimately death. One of the characteristics of DMD is muscle inflammation, and dystrophin-deficient skeletal muscles produce higher levels of the pro-inflammatory cytokine interleukin 1β (IL-1β) in response to toll like receptor (TLR) stimulation compared to controls; therefore, blocking the IL-1β pathway could improve the disease phenotype in *mdx* mice, a mouse model of DMD. Kineret^®^ or IL-1Ra is a recombinant IL-1 receptor antagonist approved by the FDA for treating rheumatoid arthritis. To determine the efficacy of IL-1Ra in a DMD model, we administered subcutaneous injections of saline control or IL-1Ra (25 mg/kg/day) to *mdx* mice daily for 45 days beginning at 5 weeks of age. Functional and histological parameters were measured at the conclusion of the study. IL-1Ra only partially inhibited this signaling pathway in this study; however, there were still interesting observations to be noted. For example, although not significantly changed, splenocytes from the IL-1Ra-treated group secreted less IL-1β after LPS stimulation compared to control mice indicating a blunted response and incomplete inhibition of the pathway (37% decrease). In addition, normalized forelimb grip strength was significantly increased in IL-1Ra-treated mice. There were no changes in EDL muscle-specific force measurements, histological parameters, or motor coordination assessments in the dystrophic mice after IL-1Ra treatment. There was a significant 27% decrease in the movement time and total distance traveled by the IL-1Ra treated mice, correlating with previous studies examining effects of IL-1 on behavior. Our studies indicate partial blocking of IL-1β with IL-1Ra significantly altered only a few behavioral and strength related disease parameters; however, treatment with inhibitors that completely block IL-1β, pathways upstream of IL-1β production or combining various inhibitors may produce more favorable outcomes.

## Introduction

Duchenne muscular dystrophy (DMD) is an X-linked muscle disease characterized by inflammation and fibrosis in the skeletal muscles which results from constant cycles of muscle degeneration and regeneration [1–3]. DMD is a progressive muscle weakness disease which causes loss of ambulation by the teenage years and mortality by the third decade of life usually due to cardiovascular complications. Despite the severity of this disease, there are only limited treatment options for DMD patients with the current standard-of-care regimen being glucocorticoids (GCs) [4–6]. These drugs have been demonstrated to delay the onset of symptoms associated with DMD; however, GCs have many side effects in patients, highlighting the need for safer and more effective alternative therapies. The *mdx* mouse model, identified via a spontaneous mutation in the dystrophin gene, exhibits some of the hallmark pathologies of DMD [3,7,8]. In this model, inflammation develops in both the limbs and diaphragm at 3 weeks of age, with a peak at about age 8–10 weeks, before diminishing in the limbs but not the diaphragm [9]. Pre-clinical testing has demonstrated that anti-inflammatory drugs improve the *mdx* muscle phenotype and therefore have the potential to alleviate inflammatory pathways in DMD patients [10–13].

Inflammatory cytokines play a major role in the DMD phenotype and these include factors like tumor necrosis factor alpha (TNFα) and interleukin 1 beta (IL-1β). Expression of TNFα in *mdx* mice has been well characterized and shown to be increased with age in the diaphragm muscle where inflammation is usually high in this model [14]. Many studies have been performed to block this signaling at various levels to improve the dystrophic phenotype by reducing necrosis, degeneration and contraction-induced injury [14–22]. Because TNFα had already been examined extensively, another cytokine of particular interest to target in DMD is IL-1β. It has previously been shown that IL-1β plays a role in the initiation and perpetuation of muscle pathology in both DMD and limb girdle muscular dystrophy 2B (LGMD2B) patients [23]. In addition, IL-1β mRNA levels are higher in *mdx* mice than in controls, and reducing both the expression and activity of IL-1β could potentially treat muscle inflammation [23]. IL-1β is secreted as a precursor protein and becomes biologically active after undergoing proteolytic cleavage by caspase-1 [24]. IL-1β mediates signaling via the interleukin 1 receptor (IL-1R) and downstream activation of the nuclear factor kappa B (NFκB) pathway. Interestingly, NFκB activity has been previously shown to be elevated in the muscle of *mdx* mice [23]. Conversely, blocking NFκB activity has been shown to reduce the inflammatory response and IL-1β levels in both DMD patients and *mdx* mice [11,12,25,26]. These effects are similar to those seen in patients on GCs and can potentially be used in a combinatorial manner to reduce muscle inflammation even further [5,6,27–31].

The interleukin 1 receptor antagonist (IL-1Ra) is a naturally occurring cytokine that inhibits the binding of IL-1β to IL-1R. IL-1Ra lacks the binding domain necessary for recruiting the IL-1R accessory protein to the receptor complex; therefore, preventing downstream pro-inflammatory signaling. A synthetic form of IL-1Ra, anakinra (Kineret^®^), is a recombinant and non-glycosylated form of human IL-1Ra that has been granted approval for use in arthritis by the Food and Drug Administration (FDA). Anakinra exerts its physiological effects in a similar manner to the naturally occurring antagonist, by competitively binding to the IL-1R and neutralizing the effects of IL-1β. The protective role of anakinra in many diseases, including those affected by inflammation, makes this compound attractive for the treatment of inflammatory diseases of the muscle, such as myositis and DMD. Since inflammation plays a detrimental role in DMD, the high level of IL-1β in the muscles of DMD patients and *mdx* mice make this pathway an attractive target for reducing the muscle pathology in these affected individuals. We hypothesized that the inflammatory effects of DMD would be reduced by treatment with anakinra and that, *in vivo*, *mdx* mice would have improved muscle function.

## Materials and Methods

### Animal care

All animal work was conducted in accordance with guidelines for the care and use of laboratory animals provided by the National institutes of health and protocols were approved by the Children’s National Institutional Animal Care and Use Committee (IACUC) (Protocol #304-13-04). For surgery, all animals were anesthetized using ketamine/xylazine and euthanasia was performed while under anesthesia using cervical dislocation. Four-week-old female C57BL/10ScSn-Dmd^*mdx*^/J (*mdx*) mice weighing 10–18 g were purchased from The Jackson Laboratory (Bar Harbor, ME). Mice were housed in individually ventilated cage system with a 12-h light-dark cycle and received standard mouse chow and water *ad libitum*. Mice were rested at least 7 days before treatment and treatment began when the mice were 5 weeks old. All functional measures were acquired in a blinded manner.

### Study design

IL-1Ra (anakinra, trade name Kineret^®^) was a gift from Amgen Inc. (Thousand Oaks, CA). Our study involved daily subcutaneous injections of two groups of animals: (a) a control *mdx* group, dosed with 0.9% NaCl (n = 6), and (b) a drug-treated *mdx* group, which received IL-1Ra at 25 mg/kg/day in a 50-μL volume of 0.9% NaCl (n = 10). Mice were randomized on the basis of body mass and were treated for 45 days, beginning at 5 weeks of age ending when they were 12 weeks old. IL-1Ra and the vehicle were injected subcutaneously, 7 days a week.

### Enzyme-linked immunosorbent assay (ELISA)

Primary splenocytes were isolated at necropsy from *mdx* mice that had been treated with IL-1Ra or saline; the cells were then maintained for 24 h *in vitro*. The medium was changed, and the splenocytes were treated for 24 h with lipopolysaccharide (LPS) or LPS-free medium. IL-1 was measured in the medium from the splenocytes. IL-1 was quantified using a Quantikine IL-1 ELISA kit (R&D Systems, Minneapolis, MN) according to the manufacturer’s instructions. N = 4 for each group analyzed.

### Rotarod test

Rotarod tests were performed as described previously [32,33]. In brief, mice were trained for 2 days prior to being tested twice a day for 3 consecutive days according to the following parameters: 10 rpm for 60s (the stabilization period), followed by acceleration from 10 rpm to 40 rpm (reached within the first 25s), totaling 240 s. Latency to fall (in seconds, s) was recorded, and six scores were averaged for each mouse. N = 6 saline, n = 10 IL-1Ra treated.

### Grip strength testing

Grip strength was assessed using a grip strength meter as previously described [26,33]. Five successful hindlimb and forelimb strength measurements within 2 min were recorded. The maximum values for each day over a 5-day period were used for subsequent analysis, and the data were normalized to body mass and expressed as kilogram force (KGF). N = 6 saline, n = 10 IL-1Ra treated.

### *In vitro* force measurements

Maximal force (mN) and twitch force (mN) generated by the extensor digitorum longus (EDL) muscle from the right hind limb was measured using a force transducer (Aurora Scientific, Aurora, Ontario, Canada) as previously described [11]. Specific force was calculated by dividing the maximal force generated by the EDL muscle by the cross-sectional area of the muscle (kN/m^2^). N = 4 saline, n = 6 IL-1Ra treated.

### Histological evaluations

Mice were anesthetized using ketamine followed by cervical dislocation in order to prevent animal suffering. Muscles were harvested and a portion of each dissected muscle (e.g., gastrocnemius, diaphragm, EDL, or heart) was placed in formalin for paraffin embedding. These tissues were later sectioned and stained with hematoxylin and eosin (H&E). The remaining portion of each tissue was embedded in Tissue-Tek optimal cutting temperature (O.C.T.) compound, (Sakura Finetek USA, Torrance, CA) and frozen in liquid nitrogen chilled isopentane for cryosectioning. Tissues were imaged under a light microscope with a 20X objective, and a digital image was obtained using computer software (Olympus C.A.S.T. Stereology System, Olympus America Inc., Center Valley, PA). The digital images were loaded into Image J (NIH) with additional plug-ins to count the cells. The total number of cells, centralized nuclei, peripheral nuclei, and cells with centralized nuclei were counted and analyzed for comparison between treatment groups. Fibers showing degeneration (as defined by a loss of striations and a homogenous appearance of the fiber contents), regeneration (as defined by a basophilic cytoplasm and large peripheral or central nuclei with prominent nucleoli), and inflammatory foci per field were assessed in a blinded fashion as described previously [33].

### Behavioral activity measurement

Open-field activity was measured using an open-field Digiscan apparatus (Omnitech Electronics, Columbus, OH) as described previously [33]. In brief, all mice were acclimated for 60 min daily in the week prior to data collection. Data were collected every 10 min over a 1-h period each day for 4 consecutive days. Results were calculated as mean ± standard error of the mean (SEM) of all recordings. N = 6 saline, n = 10 IL-1Ra treated.

### Statistical analysis

Statistical analyses comparing two groups at a time were performed using parametric, unpaired, two-tailed, t-tests (Graph Pad, Prism software). P-values ≤ 0.05 were considered statistically significant. Values in the graphs in the figures represent Values in the graphs represent mean ± standard error of the mean (SEM).

## Results

### IL-1Ra blunted the effect of LPS on IL-1 secretion in splenocytes from *mdx* mice

**Fig 1:**
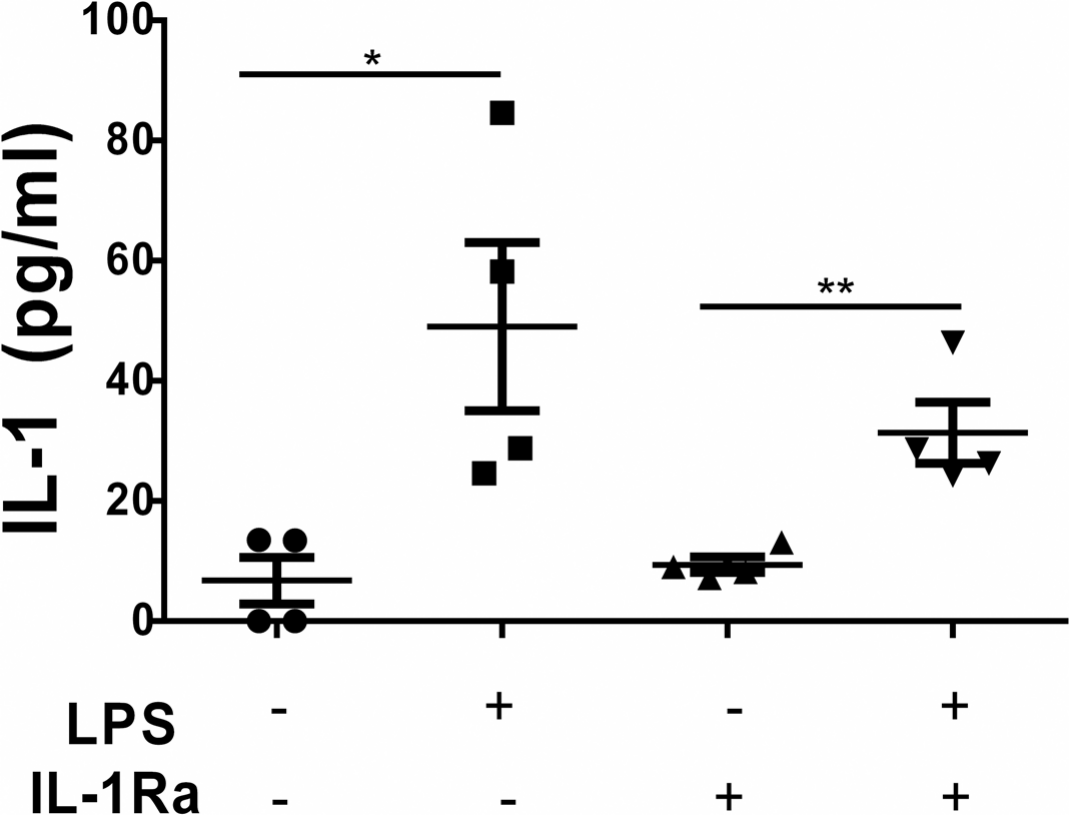
IL-1Ra blunted the effect of LPS on IL-1 secretion in splenocytes from *mdx* mice. Enzyme-linked immunosorbent assay (ELISA) was performed on medium from primary splenocytes isolated from *mdx* mice that had been treated with IL-1Ra or saline. Splenocytes from IL-1Ra- and saline-treated mice were isolated and stimulated with lipopolysaccharide (LPS). Medium was collected after 24 h to quantify the levels of IL-1 secreted into the medium by the splenocytes. LPS treatment significantly increased the IL-1 production in the splenocytes from control mice (groups 1–2) and from the IL-1Ra-treated mice (groups 3–4). Although not significant, this increase was blunted in the IL-1Ra and was 36% lower than the amount of secreted IL-1 in group 2. Values in the graphs represent mean ± SEM. Statistically significant differences were determined by using parametric, unpaired, two-tailed, t-tests with a p≤0.05 being significant (n = 4 for each group tested).

To test whether IL-1Ra is effective *in vivo* in inhibiting IL-1 secretion, splenocytes from *mdx* mice treated with IL-1Ra or saline (control) were stimulated with lipopolysaccharide (LPS). The IL-1 secretion was comparable in the unstimulated splenocytes from the saline-treated mice and the IL-1Ra-treated mice, indicating that IL-1Ra treatment alone did not alter the basal level of IL-1 secretion in the *mdx* mice (Fig 1). As expected, LPS stimulation significantly increased IL-1 secretion in saline-treated *mdx* mice. There was also a significant increase in IL-1Ra-treated splenocytes challenged with LPS. Although it was not statistically significant, it is important to note there was 37% decrease in IL-1 secretion from LPS-stimulated splenocytes of IL-1Ra-treated *mdx* mice when compared to those from saline-treated control *mdx* mice (Fig 1). Therefore, LPS-induced IL-1 secretion was blunted in the splenocytes from mice treated with IL-1Ra when compared to those receiving saline indicating IL-1Ra only partially inhibited this pathway.

### IL-1Ra treatment improves limb muscle strength in *mdx* mice

**Fig 2:**
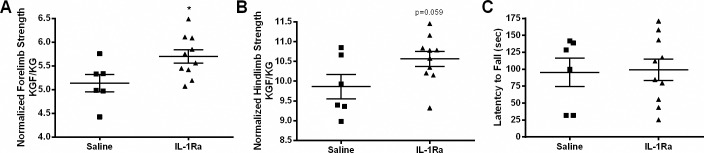
Grip strength is increased in *mdx* mice treated with IL-1Ra. Maximal strength (KGF) was measured using a grip-strength meter and normalized to body mass in kg to determine the force per kg of animal mass (KGF/KG). Animals treated with IL-1Ra (n = 10) had significantly higher (A) maximal (p = 0.047, 11% increase) and (C) normalized forelimb grip-strength (p = 0.028, 11% increase) than did control saline-treated (n = 6) animals. Hindlimb strength was measured in the same way as forelimb, and there was an increase in (B) maximal hindlimb grip strength (p = 0.100, 7% increase) and (D) normalized hindlimb strength when the means of the IL-1Ra treated (10.56 KGF/KG) and saline-injected mice (9.87 KGF/KG) were compared; this difference almost reached statistical significance (p = 0.058). There were also no significant differences in hindlimb grip strength without normalization, although a similar trend remained. (E) There were no significant differences in the latency to fall in control- (95.44s) and IL-1Ra- (99.15s) treated *mdx* mice, although the mean values showed a longer latency to fall for the IL-1Ra-treated mice. Values in the graphs represent mean ± SEM. Statistically significant differences were determined by parametric, unpaired, two-tailed, t-tests with a p≤0.05 being significant.

Grip strength measurements, performed to measure muscle strength, demonstrated that IL-1Ra-treated *mdx* mice showed a significant 11% increase in maximum forelimb strength measurements and a 7% increase in maximum hindlimb strength where statistical significance was not attained likely due to one outlier (Fig 2A and 2B). Mice treated with IL-1Ra exhibited significantly improved normalized forelimb grip strength (Fig 2C) and improvements in normalized hindlimb strength when compared to control animals (Fig 2D). The normalized forelimb and hindlimb grip strengths increased by approximately 11% (p<0.05) and 7% (p = 0.056), respectively, in the mice treated with IL-1Ra when compared to the saline-injected controls (Fig 2C and 2D). Latency to fall measurements are an indicator of motor coordination, learning, and balance; however, there were no significant differences in these measurements between IL-1Ra-treated and control *mdx* mice (Fig 2E).

### IL-1Ra treatment does not alter *in vitro* force generation or histological parameters in *mdx* mice

**Fig 3:**
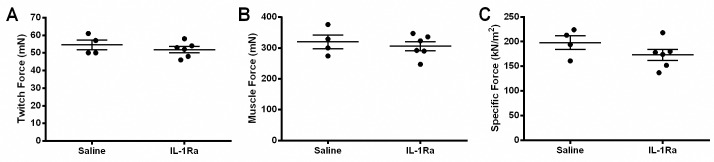
IL-1Ra treatment does not alter EDL muscle force. Muscle force in the extensor digitorum longus (EDL) muscle did not differ in saline-treated and IL-1Ra-treated *mdx* mice. There was a decrease in the strength capacity of the mice treated with IL-1Ra when compared to the controls for all measurements recorded: (A) twitch force, mN (5% decrease), (B) muscle force, mN (4% decrease) and (C) specific force, kN/m^2^ (12% decrease); however, none of these differences was statistically significant. Values in the graphs represent mean ± SEM. Statistical significance was determined by parametric, unpaired, two-tailed, t-tests (n = 4 saline and n = 6 IL-1Ra).

**Fig 4:**
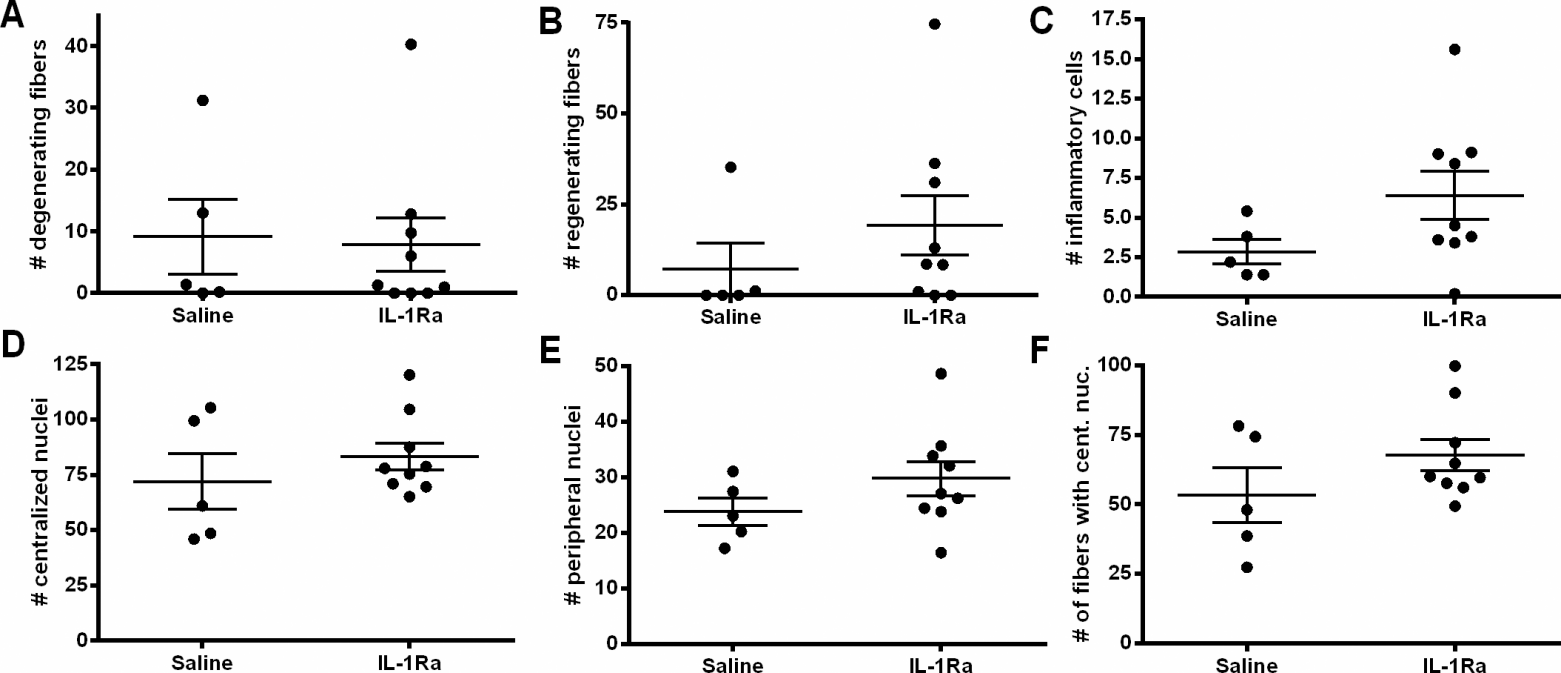
IL-1Ra treatment does not alleviate muscle pathology in *mdx* mice. Degenerating (A) and regenerating (B) muscle fibers were quantified in gastrocnemius muscle. Inflammatory cells were also quantified in sections from saline- and IL-1Ra-treated mice (C). Nuclei from sections were scored and quantified as either centralized (D) or peripheral (E). The number of fibers with centralized nuclei (F) was quantified by treatment group Values in the graphs represent mean ± SEM. Statistical significance was determined by parametric, unpaired, two-tailed, t-tests (N = 5, saline; N = 9, IL-1Ra).

To further examine muscle strength, *in vitro* force measurements were performed. The *in vitro* twitch force (mN) and absolute force (mN) of the EDL muscle did not differ between IL-1Ra-treated and saline-treated *mdx* mice (Fig 3A–3C). The specific force (kN/m^2^) and EDL mass (mg) was not significantly changed but there was a 12% reduction in specific force comparing the mean values of the IL-1Ra-treated animals which could partially be explained by the slight (not statistically significant) differences in mass of the EDL from the IL-1Ra-treated mice versus saline (9.67 mg versus 9.36 mg, Table 1). There were no significant differences in the absolute body mass, skeletal muscle (gastrocnemius, soleus) mass, or heart mass between the saline-treated and IL-1Ra-treated mice (Table 1). From histology, no differences were observed in the number of degenerating or regenerating fibers, inflammatory cells, or centralized or peripheral nuclei in the gastrocnemius muscles from the IL-1Ra-treated and control *mdx* mice (Fig 4A and 4B).

**Table 1:** Body and muscle mass in *mdx* mice after treatment with saline or IL-1Ra.

Parameters	Saline-treated	IL-1Ra-treated	*p*-value
Body mass, g	20.93 (0.42)	21.85 (0.31)	0.12
Gastrocnemius, mg	109.28 (5.06)	114.67 (3.00)	0.36
Soleus, mg	7.53 (0.42)	7.81 (0.29)	0.60
EDL, mg	9.36 (0.59)	9.67 (0.35)	0.66
Heart, mg	100.55 (5.73)	100.29 (2.68)	0.96

There were no statistically significant differences in the overall body mass or the masses of the gastrocnemius, soleus, extensor digitorum longus (EDL), or heart between the IL-1Ra-treated and control saline-treated mice. Muscle mass was expressed as the average of the left and right muscle, where applicable, and also as a percentage of the total body weight. Values are expressed as mean, with SEM in parentheses. No significant differences were determined as calculated by parametric, unpaired, two-tailed, t-tests; p-values are indicated.

### IL-1Ra treatment decreases activity in *mdx* mice

**Fig 5:**
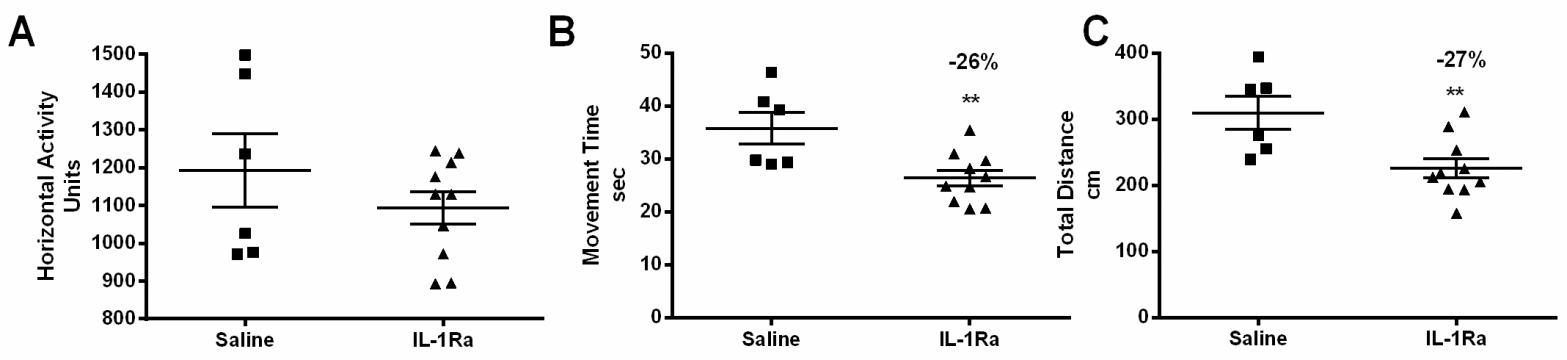
IL-1Ra treatment decreases activity and increases rest time in *mdx* mice. Horizontal activity (A) was measured using an open-field Digiscan apparatus and did not differ significantly between IL-1Ra- and saline-treated mice. During the Digiscan measurements, the (B) movement time (26% decrease in the IL-1Ra-treated mice vs. control, p = 0.0075) and (C) total distance traveled (27% decrease in the IL-1Ra-treated mice vs. control, p = 0.008) were recorded and were significantly lower in the IL-1Ra-treated mice than in the controls. Graphs represent mean ± SEM and statistically significant differences were determined by parametric, unpaired, two- tailed, t-tests with a p≤0.05 being significant. (n = 6 saline, n = 10 IL-1Ra).

Behavioral measurements of horizontal and vertical activity were performed using an open-field Digiscan apparatus as previously described [33]. IL-1Ra treatment did not have a significant effect on either horizontal or vertical activity in IL-1Ra-treated *mdx* mice when compared to saline-injected mice (Fig 5; data not shown). Movement time and total distance traveled were significantly decreased (by 27%) in the IL-1Ra-treated mice when compared to the saline-treated mice (Fig 5B and 5C).

## Discussion

During inflammatory processes, IL-1β is produced by several cell types in response to activation of a variety of innate immune receptors, including the toll-like receptor (TLR) superfamily. Stimulation of IL-1β and subsequent binding to the IL-1R activates the pro-inflammatory NFκB pathway. IL-1β signaling is controlled by IL-1Ra, a natural antagonist of IL-1R, and overexpression of IL-1β has been implicated in the pathology of a wide variety of human diseases associated with chronic inflammation. Development of inhibitors such as anakinra have been shown to be effective in many diseases with an inflammatory component including: inhibiting apoptotic events during experimental acute myocardial infarction [34], improving symptoms of type 2 diabetes [35–38] and reducing the symptoms of rheumatoid arthritis (RA) in clinical trials [39,40]. Improvements in auto-inflammatory diseases, including Muckle-Wells syndrome (MWS) [41] and gout [42] have also been described after using anakinra. Since inflammation is a major player in DMD, the IL-1β pathway is expected to play a role in the initiation and perpetuation of the muscle pathology in DMD. Muscle inflammation and necrosis is also evident in the *mdx* mouse model; therefore, IL-1Ra treatment in pre-clinical studies could prevent the inflammatory effects of systemic IL-1β secretion on IL-1R activation potentially limiting cellular inflammation. In addition, it is known that in the *mdx* mouse model of DMD, there is an increase in the expression of TNF-α and IL-1β prior to disease onset [23]. IL-1Ra is already FDA-approved making this drug a good candidate to try to inhibit IL-1β upregulation and prevent the associated downstream signaling.

To date, many preclinical studies have focused on pharmacological treatments to inhibit TNF-α as a potential treatment for DMD. Several of the drugs tested have been shown to ameliorate the muscle pathologies associated with the *mdx* phenotype [14–22]. In the present study, inhibiting IL-1β in the *mdx* model was tested using a synthetic inhibitor of IL-1R, IL-1Ra, treating *mdx* mice for 45 days. The dosing regimen selected here, 25mg/kg daily, was based on a previous study that demonstrated efficacy in a model of alcoholic steatohepatitis in mice [43]. To assess whether IL-1Ra had engaged the target, we performed an *in vitro* stimulation assay using LPS to activate splenocytes collected from treated and untreated animals. Here, we saw a blunted effect of IL-1Ra on splenocytes treated with LPS (27% less than control LPS-stimulated splenocytes), indicating IL-1Ra incompletely inhibited IL-1 under our conditions, thus allowing signaling to persist even after treatment (Fig 1).

In addition to examining signaling effects of IL-1Ra, strength testing was also performed and revealed significantly improved forelimb gripstrength and a trend for hindlimb gripstrength, though not significant, toward increasing (Fig 2). No changes were detected in latency to fall (Fig 2E), or muscle force (twitch, maximal, or specific force, Fig 3). Open-field activity measures were mainly down-regulated in the mice treated with IL-1Ra (Fig 5). One possible explanation for the 27% decrease in movement in the IL-1Ra-treated mice could be that the mice are nauseous and therefore less likely to move about the cage. This is certainly reasonable to consider since this is one of the potential side effects of IL-1Ra in humans (Fig 5). Increased gripstrength and decreased open-field activity measures could have been caused by differential effects of IL-1 signaling. For example, it is known that immobilization stress induces IL-1β in rats [44,45], IL-1 administration induces stress [46], exposure to acute stress induces IL-1 expression in rodents [47]. IL-1R-null mice show decreased anxiety, and conversely IL-1Ra-null mice show increased anxiety as they age [48,49]. Additionally, mice with IL-1β overexpression specifically in the hippocampus (IL-1β^XAT^) demonstrate increased locomotion, the converse of what was seen in these studies inhibiting that pathway [50]. IL-1 could be released to mediate a stress response, and when the natural inhibitor of IL-1, IL-1Ra, is genetically removed, the animals elicit an increased stress response [49]. For this study, the mice were handled prior to the grip strength measurement, and although they had become acclimated during the week before the measurements were collected, grip strength could have been improved because of increases in IL-1β leading to behavioral stress response. True muscle force is measured by the *in vitro* force measurements, because these measurements are not confounded by volition. *In vitro* force was not changed in our study after IL-1Ra treatment, indicating that IL-1Ra did not improve muscle strength possibly due do an incomplete inhibition of this pathway.

The skeletal muscles from the mice treated with IL-1Ra had no differences in histological measurements including inflammatory foci, muscle fibers with centralized nuclei, regenerating fibers, or degenerating fibers when compared to controls. This can also be explained by IL-1Ra being a weak inhibitor of the pathway (Fig 4). There are several other factors that could provide an explanation for this including: lack of potency, dosage, duration of treatment, and the role of the IL-1 pathway in muscle disease progression at the time of treatment. A longer-term study could reveal improved cellular patterning in IL-1Ra-treated *mdx* mice and indicate that IL-1Ra can indeed diminish the secretion of IL-1; however, this possibility still needs to be tested. For this study, treatment was administered after the onset of skeletal muscle necrosis in *mdx* mice and others have shown that inhibiting another inflammatory cytokine, TNFα, using Remicade^®^ (an anti-TNFα antibody) at seven days of age delayed onset of acute necrosis at 21 days in *mdx* mice [22]. Treating mice at earlier time points with IL-1Ra could be considered for future experiments to examine the full potential of this drug at all stages of the disease phenotype in the *mdx* mice. Interestingly, at 12 weeks there were no significant pathological changes found after Remicade^®^ treatment as we have shown after IL-1Ra treatment [22]. To overcome this challenge with the stabilized *mdx* muscle phenotype, older *mdx* mice could be treated with IL-1Ra and challenged using a treadmill to exacerbate the disease phenotype at this stage and examine additional effects from this treatment on dystrophic muscles similarly to what was done with TNFα inhibition [20]. Lastly, it is possible that inhibition of TNFα could be more efficacious than IL-1Ra treatment in the *mdx* mice; however, considerations have to be taken for examining cardiac function as long-term TNFα inhibition has been demonstrated to have a negative impact on heart function [51].

Overall, the dose of IL-1Ra used here may have been enough to elicit a behavioral response but insufficient to completely block the inflammatory signaling cascade and associated phenotype in the skeletal muscles of the *mdx* mice. In order to further examine the inhibition of IL-1 and the benefits of down-regulating the secretion of this inflammatory factor, other IL-1R inhibitors could also be examined in preclinical trials. They could be compared with IL-1Ra to determine whether or not they are more potent inhibitors and, if so, what histological and functional outcomes might improve from their use. Others have examined the possibility of decreasing inflammation by using exon-skipping technology targeting IL-1RAcP [52]. Targeting IL-1R can have broad applications for many inflammatory diseases, and the mechanisms of its inhibition are important to understand in order to decrease inflammation associated with the disease. The goal of the ongoing studies in our laboratory is to broaden the therapeutic options for DMD patients and their families beyond prolonged steroid regimens. In this short-term study of *mdx* mice, which have a milder phenotype than DMD patients, IL-1Ra treatment displayed an improvement in some functional parameters for muscle strength when compared to saline-treated mice; however, these changes could have been a result of the behavioral effects of the treatment. Optimization of the dosage and timing of the IL-1Ra treatment is clearly needed to clarify whether a greater inhibition of the IL-1β signaling pathway (and resulting clinical improvement) can be achieved in DMD.

## References

[1] Hoffman EP, Brown RH Jr., Kunkel LM (1987) Dystrophin: the protein product of the Duchenne muscular dystrophy locus. Cell 51: 919–928. 3319190 10.1016/0092-8674(87)90579-4

[2] Hoffman EP, Knudson CM, Campbell KP, Kunkel LM (1987) Subcellular fractionation of dystrophin to the triads of skeletal muscle. Nature 330: 754–758. 2447503 10.1038/330754a0

[3] Hoffman EP, Monaco AP, Feener CC, Kunkel LM (1987) Conservation of the Duchenne muscular dystrophy gene in mice and humans. Science 238: 347–350. 3659917 10.1126/science.3659917

[4] Manzur AY, Kinali M, Muntoni F (2008) Update on the management of Duchenne muscular dystrophy. Arch Dis Child 93: 986–990. 10.1136/adc.2007.118141 18667451

[5] Manzur AY, Kuntzer T, Pike M, Swan A (2008) Glucocorticoid corticosteroids for Duchenne muscular dystrophy. Cochrane Database Syst Rev: CD003725 10.1002/14651858.CD003725.pub3 18254031

[6] Markham LW, Kinnett K, Wong BL, Woodrow Benson D, Cripe LH (2008) Corticosteroid treatment retards development of ventricular dysfunction in Duchenne muscular dystrophy. Neuromuscular disorders: NMD 18: 365–370. 10.1016/j.nmd.2008.03.002 18436445

[7] Bulfield G, Siller WG, Wight PA, Moore KJ (1984) X chromosome-linked muscular dystrophy (mdx) in the mouse. Proceedings of the National Academy of Sciences of the United States of America 81: 1189–1192. 6583703 10.1073/pnas.81.4.1189 PMC344791

[8] Grounds MD, Radley HG, Lynch GS, Nagaraju K, De Luca A (2008) Towards developing standard operating procedures for pre-clinical testing in the mdx mouse model of Duchenne muscular dystrophy. Neurobiol Dis 31: 1–19. 10.1016/j.nbd.2008.03.008 18499465 PMC2518169

[9] Tanabe Y, Esaki K, Nomura T (1986) Skeletal muscle pathology in X chromosome-linked muscular dystrophy (mdx) mouse. Acta neuropathologica 69: 91–95. 3962599 10.1007/BF00687043

[10] Huang P, Zhao XS, Fields M, Ransohoff RM, Zhou L (2009) Imatinib attenuates skeletal muscle dystrophy in mdx mice. FASEB journal: official publication of the Federation of American Societies for Experimental Biology 23: 2539–2548. 19289603 10.1096/fj.09-129833 PMC2717779

[11] Heier CR, Damsker JM, Yu Q, Dillingham BC, Huynh T, Van der Meulen JH, et al (2013) VBP15, a novel anti-inflammatory and membrane-stabilizer, improves muscular dystrophy without side effects. EMBO Mol Med 5: 1569–1585. 10.1002/emmm.201302621 24014378 PMC3799580

[12] Messina S, Bitto A, Aguennouz M, Minutoli L, Monici MC, Altavilla D, et al (2006) Nuclear factor kappa-B blockade reduces skeletal muscle degeneration and enhances muscle function in Mdx mice. Experimental neurology 198: 234–241. 16410003 10.1016/j.expneurol.2005.11.021

[13] Tang Y, Reay DP, Salay MN, Mi MY, Clemens PR, Guttridge DC, et al (2010) Inhibition of the IKK/NF-kappaB pathway by AAV gene transfer improves muscle regeneration in older mdx mice. Gene Ther 17: 1476–1483. 10.1038/gt.2010.110 20720575 PMC3471137

[14] Barros Maranhao J, de Oliveira Moreira D, Mauricio AF, de Carvalho SC, Ferretti R, Pereira JA, et al (2015) Changes in calsequestrin, TNF-alpha, TGF-beta and MyoD levels during the progression of skeletal muscle dystrophy in mdx mice: a comparative analysis of the quadriceps, diaphragm and intrinsic laryngeal muscles. Int J Exp Pathol 96: 285–293. 10.1111/iep.12142 26515458 PMC4693553

[15] Pierno S, Nico B, Burdi R, Liantonio A, Didonna MP, Cippone V, et al (2007) Role of tumour necrosis factor alpha, but not of cyclo-oxygenase-2-derived eicosanoids, on functional and morphological indices of dystrophic progression in mdx mice: a pharmacological approach. Neuropathol Appl Neurobiol 33: 344–359. 17493014 10.1111/j.1365-2990.2007.00798.x

[16] de Senzi Moraes Pinto R, Ferretti R, Moraes LH, Neto HS, Marques MJ, Minatel E (2013) N-acetylcysteine treatment reduces TNF-alpha levels and myonecrosis in diaphragm muscle of mdx mice. Clin Nutr 32: 472–475. 10.1016/j.clnu.2012.06.001 22727548

[17] Machado RV, Mauricio AF, Taniguti AP, Ferretti R, Neto HS, Marques MJ (2011) Eicosapentaenoic acid decreases TNF-alpha and protects dystrophic muscles of mdx mice from degeneration. J Neuroimmunol 232: 145–150. 10.1016/j.jneuroim.2010.10.032 21131061

[18] Piers AT, Lavin T, Radley-Crabb HG, Bakker AJ, Grounds MD, Pinniger GJ (2011) Blockade of TNF in vivo using cV1q antibody reduces contractile dysfunction of skeletal muscle in response to eccentric exercise in dystrophic mdx and normal mice. Neuromuscul Disord 21: 132–141. 10.1016/j.nmd.2010.09.013 21055937

[19] Waters FJ, Shavlakadze T, McIldowie MJ, Piggott MJ, Grounds MD (2010) Use of pifithrin to inhibit p53-mediated signalling of TNF in dystrophic muscles of mdx mice. Mol Cell Biochem 337: 119–131. 10.1007/s11010-009-0291-2 19859789

[20] Radley HG, Davies MJ, Grounds MD (2008) Reduced muscle necrosis and long-term benefits in dystrophic mdx mice after cV1q (blockade of TNF) treatment. Neuromuscul Disord 18: 227–238. 10.1016/j.nmd.2007.11.002 18207402

[21] Grounds MD, Davies M, Torrisi J, Shavlakadze T, White J, Hodgetts S (2005) Silencing TNFalpha activity by using Remicade or Enbrel blocks inflammation in whole muscle grafts: an in vivo bioassay to assess the efficacy of anti-cytokine drugs in mice. Cell Tissue Res 320: 509–515. 15846506 10.1007/s00441-005-1102-z

[22] Grounds MD, Torrisi J (2004) Anti-TNFalpha (Remicade) therapy protects dystrophic skeletal muscle from necrosis. FASEB J 18: 676–682. 15054089 10.1096/fj.03-1024com

[23] Rawat R, Cohen TV, Ampong B, Francia D, Henriques-Pons A, Hoffman EP, et al (2010) Inflammasome up-regulation and activation in dysferlin-deficient skeletal muscle. The American journal of pathology 176: 2891–2900. 10.2353/ajpath.2010.090058 20413686 PMC2877850

[24] Dinarello CA (1996) Biologic basis for interleukin-1 in disease. Blood 87: 2095–2147. 8630372

[25] Messina S, Altavilla D, Aguennouz M, Seminara P, Minutoli L, Monici MC, et al (2006) Lipid peroxidation inhibition blunts nuclear factor-kappaB activation, reduces skeletal muscle degeneration, and enhances muscle function in mdx mice. The American journal of pathology 168: 918–926. 16507907 10.2353/ajpath.2006.050673 PMC1606515

[26] Huynh T, Uaesoontrachoon K, Quinn JL, Tatem KS, Heier CR, Van Der Meulen JH, et al (2013) Selective modulation through the glucocorticoid receptor ameliorates muscle pathology in mdx mice. J Pathol 231: 223–235. 10.1002/path.4231 23794417 PMC4104819

[27] Angelini C, Pegoraro E, Turella E, Intino MT, Pini A, Costa C (1994) Deflazacort in Duchenne dystrophy: study of long-term effect. Muscle & nerve 17: 386–391. 8170484 10.1002/mus.880170405

[28] Biggar WD, Harris VA, Eliasoph L, Alman B (2006) Long-term benefits of deflazacort treatment for boys with Duchenne muscular dystrophy in their second decade. Neuromuscular disorders: NMD 16: 249–255. 16545568 10.1016/j.nmd.2006.01.010

[29] Griggs RC, Moxley RT 3rd, Mendell JR, Fenichel GM, Brooke MH, Pestronk A, et al (1991) Prednisone in Duchenne dystrophy. A randomized, controlled trial defining the time course and dose response. Clinical Investigation of Duchenne Dystrophy Group. Arch Neurol 48: 383–388. 2012511 10.1001/archneur.1991.00530160047012

[30] Griggs RC, Moxley RT 3rd, Mendell JR, Fenichel GM, Brooke MH, Pestronk A, et al (1993) Duchenne dystrophy: randomized, controlled trial of prednisone (18 months) and azathioprine (12 months). Neurology 43: 520–527. 8450994 10.1212/wnl.43.3_part_1.520

[31] Mendell JR, Moxley RT, Griggs RC, Brooke MH, Fenichel GM, Miller JP, et al (1989) Randomized, double-blind six-month trial of prednisone in Duchenne's muscular dystrophy. The New England journal of medicine 320: 1592–1597. 2657428 10.1056/NEJM198906153202405

[32] Aro AA, Freitas KM, Foglio MA, Carvalho JE, Dolder H, Gomes L, et al (2013) Effect of the Arrabidaea chica extract on collagen fiber organization during healing of partially transected tendon. Life sciences 92: 799–807. 10.1016/j.lfs.2013.02.011 23454166

[33] Spurney CF, Gordish-Dressman H, Guerron AD, Sali A, Pandey GS, Rawat R, et al (2009) Preclinical drug trials in the mdx mouse: assessment of reliable and sensitive outcome measures. Muscle & nerve 39: 591–602. 19260102 10.1002/mus.21211 PMC4116326

[34] Abbate A, Salloum FN, Vecile E, Das A, Hoke NN, Straino S, et al (2008) Anakinra, a recombinant human interleukin-1 receptor antagonist, inhibits apoptosis in experimental acute myocardial infarction. Circulation 117: 2670–2683. 10.1161/CIRCULATIONAHA.107.740233 18474815

[35] Mandrup-Poulsen T, Zumsteg U, Reimers J, Pociot F, Morch L, Helqvist S, et al (1993) Involvement of interleukin 1 and interleukin 1 antagonist in pancreatic beta-cell destruction in insulin-dependent diabetes mellitus. Cytokine 5: 185–191. 8218929 10.1016/1043-4666(93)90003-n

[36] Koivisto VA, Leirisalo-Repo M, Ebeling P, Tuominen JA, Knip M, Turunen U, et al (1993) Seven years of remission in a type I diabetic patient. Diabetes care 16: 990–995. 8359107 10.2337/diacare.16.7.990

[37] Larsen CM, Faulenbach M, Vaag A, Ehses JA, Donath MY, Mandrup-Poulsen T (2009) Sustained effects of interleukin-1 receptor antagonist treatment in type 2 diabetes. Diabetes care 32: 1663–1668. 10.2337/dc09-0533 19542207 PMC2732140

[38] Lacraz G, Giroix MH, Kassis N, Coulaud J, Galinier A, Noll C, et al (2009) Islet endothelial activation and oxidative stress gene expression is reduced by IL-1Ra treatment in the type 2 diabetic GK rat. PloS one 4: e6963 10.1371/journal.pone.0006963 19742300 PMC2737103

[39] Mertens M, Singh JA (2009) Anakinra for rheumatoid arthritis: a systematic review. J Rheumatol 36: 1118–1125. 10.3899/jrheum.090074 19447938

[40] Bresnihan B, Alvaro-Gracia JM, Cobby M, Doherty M, Domljan Z, Emery P, et al (1998) Treatment of rheumatoid arthritis with recombinant human interleukin-1 receptor antagonist. Arthritis and rheumatism 41: 2196–2204. 9870876 10.1002/1529-0131(199812)41:12<2196::AID-ART15>3.0.CO;2-2

[41] Dalgic B, Egritas O, Sari S, Cuisset L (2007) A variant Muckle-Wells syndrome with a novel mutation in CIAS1 gene responding to anakinra. Pediatric nephrology 22: 1391–1394. 17486372 10.1007/s00467-007-0500-8

[42] So A, De Smedt T, Revaz S, Tschopp J (2007) A pilot study of IL-1 inhibition by anakinra in acute gout. Arthritis research & therapy 9: R28. 17352828 10.1186/ar2143 PMC1906806

[43] Petrasek J, Bala S, Csak T, Lippai D, Kodys K, Menashy V, et al (2012) IL-1 receptor antagonist ameliorates inflammasome-dependent alcoholic steatohepatitis in mice. J Clin Invest 122: 3476–3489. 10.1172/JCI60777 22945633 PMC3461900

[44] Tringali G, Dello Russo C, Preziosi P, Navarra P (2000) Interleukin-1 in the central nervous system: from physiology to pathology. Therapie 55: 171–175. 10860021

[45] Minami M, Kuraishi Y, Yamaguchi T, Nakai S, Hirai Y, Satoh M (1991) Immobilization stress induces interleukin-1 beta mRNA in the rat hypothalamus. Neurosci Lett 123: 254–256. 2027540 10.1016/0304-3940(91)90944-o

[46] Cragnolini AB, Schioth HB, Scimonelli TN (2006) Anxiety-like behavior induced by IL-1beta is modulated by alpha-MSH through central melanocortin-4 receptors. Peptides 27: 1451–1456. 16325304 10.1016/j.peptides.2005.10.020

[47] Nguyen KT, Deak T, Owens SM, Kohno T, Fleshner M, Watkins LR, et al (1998) Exposure to acute stress induces brain interleukin-1beta protein in the rat. J Neurosci 18: 2239–2246. 9482808 10.1523/JNEUROSCI.18-06-02239.1998 PMC6792918

[48] Koo JW, Duman RS (2009) Interleukin-1 receptor null mutant mice show decreased anxiety-like behavior and enhanced fear memory. Neurosci Lett 456: 39–43. 10.1016/j.neulet.2009.03.068 19429130 PMC3678367

[49] Wakabayashi C, Numakawa T, Odaka H, Ooshima Y, Kiyama Y, Manabe T, et al (2015) IL-1 receptor-antagonist (IL-1Ra) knockout mice show anxiety-like behavior by aging. Neurosci Lett 599: 20–25. 10.1016/j.neulet.2015.05.019 26002078

[50] Hein AM, Zarcone TJ, Parfitt DB, Matousek SB, Carbonari DM, Olschowka JA, et al (2012) Behavioral, structural and molecular changes following long-term hippocampal IL-1beta overexpression in transgenic mice. J Neuroimmune Pharmacol 7: 145–155. 10.1007/s11481-011-9294-3 21748283 PMC3214690

[51] Ermolova NV, Martinez L, Vetrone SA, Jordan MC, Roos KP, Sweeney HL, et al (2014) Long-term administration of the TNF blocking drug Remicade (cV1q) to mdx mice reduces skeletal and cardiac muscle fibrosis, but negatively impacts cardiac function. Neuromuscul Disord 24: 583–595. 10.1016/j.nmd.2014.04.006 24844454 PMC4122520

[52] Yilmaz-Elis AS, Aartsma-Rus A, t Hoen PA, Safdar H, Breukel C, van Vlijmen BJ, et al (2013) Inhibition of IL-1 Signaling by Antisense Oligonucleotide-mediated Exon Skipping of IL-1 Receptor Accessory Protein (IL-1RAcP). Mol Ther Nucleic Acids 2: e66 10.1038/mtna.2012.58 23340324 PMC3564974

